# Homocysteine Levels Are Associated With the Rupture of Intracranial Aneurysms

**DOI:** 10.3389/fnins.2022.945537

**Published:** 2022-07-14

**Authors:** Sen Wei, Xin Yuan, Dongdong Li, Xinbin Guo, Sheng Guan, Yuming Xu

**Affiliations:** ^1^Department of Neurointervention, The First Affiliated Hospital of Zhengzhou University at Zhengzhou, Zhengzhou, China; ^2^Department of Neurology, The First Affiliated Hospital of Zhengzhou University at Zhengzhou, Zhengzhou, China

**Keywords:** intracranial aneurysm, rupture, risk, subarachnoid hemorrhage, homocysteine

## Abstract

**Background:**

Homocysteine (Hcy) levels may be associated with the development of intracranial aneurysms (IAs). However, whether it increases the risk of rupture of IAs is unknown. This study aimed to determine the association between homocysteine levels and IA rupture.

**Methods:**

We retrospectively reviewed patients with IAs and subarachnoid hemorrhage (SAH) at our hospital between January 2019 and May 2021. Clinical data, including Hcy levels and IA images, were assessed. The association between Hcy level and IA rupture was investigated using multivariate logistic regression analyses in patients with IAs and SAH.

**Results:**

A total of 589 patients were included. 546 patients with IAs, including 331 UIA (Unruptured IA) and 215 RIA (Ruptured IA). The average age was 57.43 ± 10.86 years old, and 67.03% were women. Among them, all 215 RIAs lead to SAH. In addition, we also enrolled 43 non-aneurysmal subarachnoid hemorrhage (Na-SAH) patients. The average age was 54.12 ± 10.55 years old, and 53.48% were female. After adjusting for confounders in the multivariate model, Hcy levels were correlated with the rupture of IA (odds ratio [OR] 1.069; 95% confidence interval [CI] 1.025–1.114, *p* = 0.002) and a-SAH (OR 1.083; 95% CI 1.002–1.170, *p* = 0.046).

**Conclusion:**

Hcy levels were associated with IA rupture. These findings provide novel insights into IAs rupture, and future studies are needed to confirm this relationship.

## Introduction

Intracranial aneurysms (IAs) are a common disease that endangers human health. The incidence of unruptured intracranial aneurysms (UIA) in the population is 1-6% (Vlak et al., 2011). Most UIA are stable, and approximately 1% of UIA cases rupture annually (Wang et al., 2021). UIA rupture accounts for approximately 85% of subarachnoid hemorrhages (SAH). The incidence of repeat bleeding within two weeks and one month was 25 and 40%, respectively, and the fatality rate of repeat bleeding was more than 40% (Tawk et al., 2021). Studies have shown that the acute rupture of a UIA has a mortality rate of 20-30% (Vergouwen et al., 2016). If bleeding occurs again, the death rate is as high as 60-80%, and many survivors are disabled (Vergouwen et al., 2016). To date, aneurysmal clipping and endovascular interventional therapy have been the primary methods for treating IAs. However, the treatment effect for aneurysmal SAH is extremely poor, and the morbidity and mortality rates remain high (Tawk et al., 2021). Therefore, it is of great clinical significance to accurately assess the risk of rupture in patients with UIA. Previous studies have focused on the effects of clinical factors of UIA patients, such as sex, age, hypertension, and aneurysmal morphological factors (size and location) (Greving et al., 2014; Fuentes et al., 2022; Wang et al., 2022). However, few studies have investigated the role of biomarkers in IA rupture.

Homocysteine (Hcy) is a sulfur-containing amino acid, an intermediate product of the methionine cycle, which the human body maintains through the dietary intake of folic acid and vitamins B6 and B12 (Selhub et al., 1993). Diets with excess methionine, folate deficiency, or genetic alterations in certain enzymes of the methionine cycle can increase Hcy levels in the body (Selhub et al., 1993; Jacques et al., 2001). It is believed that Hcy is an important biomarker of cardiovascular and cerebrovascular diseases. Hyper-homocysteinemia (HHcy) not only increases the risk of cardiovascular disease and stroke (Shoamanesh et al., 2016) but also affects the prognosis of stroke patients (Shi et al., 2015) and increases the risk of dementia (Li et al., 2014; Pařízková et al., 2017). In recent years, some studies found that Hcy was associated with the occurrence of abdominal aortic aneurysms (AAA) (Li et al., 2018; Miao et al., 2021). Observational studies also have found that Hcy is related to the development of IAs (Ren et al., 2017; Rosi et al., 2018; Wang et al., 2020). However, since the subjects of these studies include ruptured and unruptured aneurysms and the influence of SAH on Hcy levels cannot be completely excluded, it is still unknown whether Hcy increases the risk of IA rupture. This study aimed to investigate the association between Hcy levels and the rupture of IAs.

## Materials and Methods

### Subjects

We retrospectively identified patients with IA and non-aneurysmal subarachnoid hemorrhage (Na-SAH) at our hospital between January 2019 and May 2021. The following inclusion criteria were applied: (1) age ≥18 years; (2) hospitalized with a primary diagnosis of spontaneous SAH according to the World Health Organization criteria, which was confirmed by brain computerized tomography (CT) within seven days of symptom onset, or hospitalized with a primary diagnosis of IA according to the World Health Organization criteria, which was confirmed with digital subtraction angiography (DSA); (3) Hunt-Hess scale ≤3; (4) no other severe physical illnesses that were life-threatening or interfering with the stroke recovery evaluation; (5) plasma Hcy values obtained upon hospital admission. (6) supplemented folic acid or vitamin B12 drugs in recent three months; (7) Patients who did not undergo CT or DSA, or those with rare causes of SAH, such as cerebrovascular malformations and trauma, were excluded.

All IA and Na-SAH patients underwent DSA after admission. In SAH patients, the DSA was used to identify the presence or absence of IAs. Patients with IAs in the study were divided into UIA and ruptured intracranial aneurysm (RIA). Patients with SAH were divided into a-SAH and Na-SAH. Two weeks after onset, patients with SAH and an initial negative DSA were re-examined using either CT angiography (CTA) or DSA. Patients with SAH with a negative two-week CTA or DSA were re-examined using CTA three months later. SAH patients with IAs at any time point were included in the a-SAH group, and those without IAs at all examinations were included in the Na-SAH group.

### Ethics Statement

The study protocol was approved by the ethics committee of the First Affiliated Hospital of Zhengzhou University, and informed consent was obtained from all the participants or their legally authorized representatives.

### Data Collection

Based on past medical history, risk factors were ascertained via direct patient and/or proxy interviews. Lifestyle risk factors included smoking (current or past) and drinking (current or past). Hypertension was defined as at least two raised blood pressure measurements (either ≥140 mmHg systolic or ≥90 mmHg diastolic) on separate days before the stroke, the use of antihypertensive medications, or a physician’s diagnosis. Prior stroke was defined as a medically confirmed history of ischemic or hemorrhagic stroke or subarachnoid hemorrhage. Coronary artery disease (CAD) was defined as a history of angina pectoris, myocardial infarction, or the use of CAD medication. Diabetes mellitus was defined as a previous measure of a 2-h oral glucose tolerance test value ≥200 mg/dL, insulin or oral hypoglycemic medicine use, or a physician’s diagnosis. Hyperlipidemia was defined as a history of hyperlipidemia, use of lipid-lowering medications, or diagnosis by a physician. The estimated glomerular filtration rate, an indicator of baseline kidney function, was calculated using the Modification of Diet in Renal Disease formula (Levey et al., 1999). Other potential risk factors included folate, vitamin B12, creatinine, and uric acid.

### Blood Measurements

Fasting blood samples were collected in evacuated tubes containing EDTA after overnight fasting for at least 8 h. The samples were centrifuged within 1h and stored below −20°C until analysis. Hcy levels were measured using a fluorescence polarization immunoassay analyzer (Abbott Laboratories, Chicago, IL, United States).

### Digital Subtraction Angiography Imaging

Imaging sequences, including 2-dimensional conventional angiograms and 3-dimensional conventional angiograms of DSA, were used to assess aneurysmal location, shape irregularities, maximum size, and number of IAs. The irregular shape was defined as the aneurysm fundus was bi- or multi-lobular, small bleb(s), or secondary aneurysm(s) protruding from the aneurysm fundus, according to recent research (Wang et al., 2022). Two clinical stroke neurologists performed the imaging assessment blinded to the patient’s clinical data and baseline characteristics for our study. In cases where a discrepancy arose, the final decision was reached by consensus.

### Statistical Analysis

All continuous variables were expressed as means ± standard deviation, and categorical variables as frequencies and percentages. Categorical variables were analyzed by chi-square tests, whereas differences in mean values of continuous variables were assessed with Student’s *t*-test for independent samples. When the normality assumption was violated, Mann–Whitney *U*-test was used. Factors related to IAs in the univariate analyses (*P* < 0.05) were included in the multivariate model as candidate variables. All statistical analyses were performed using SPSS version 23.0. Two-tailed tests of probability (*P* < 0.05) were used to estimate statistical significance in all analyses.

## Results

### Demographic and Clinical Characteristics

The demographic and clinical characteristics of the patients are presented in Table 1. A total of 589 patients were included: 546 patients with IA, including 331 UIAs and 215 RIAs. The average age of these patients was 57.43 ± 10.86 years old, of which 67.03% were women. Among them, all 215 ruptured aneurysms lead to SAH. Therefore, these patients were also regarded as a-SAH. In addition, we also enrolled 43 Na-SAH patients. The average age of this group was 54.12 ± 10.55 years old, and 53.48% were female.

**TABLE 1:** Demographic and clinical characteristics of patients with intracranial aneurysms and subarachnoid hemorrhages.

Index	UIAs (*n* = 331)	RIAs (*n* = 215)	Na-SAH (*n* = 43)	*p1* value	*p2* value
Age, year	57.78 ± 10.14	56.89 ± 11.90	54.12 ± 10.55	0.543	0.120
Sex(female), n (%)	233(70.39%)	133(61.86%)	23(53.48%)	**0.038**	0.305
Smoking, n (%)	45(13.60%)	45(20.93%)	7(16.27%)	**0.024**	0.488
Drinking, n (%)	45(13.60%)	45(15.35%)	8(18.60%)	0.567	0.594
Hypertension, n (%)	174(52.57%)	121(56.28%)	13(30.23%)	0.395	**0.002**
Diabetes mellitus, n (%)	53(16.01%)	10(4.65%)	2(4.65%)	**<0.001**	1.000
Dyslipidemia, n (%)	102(30.82%)	60(27.91%)	12(27.91%)	0.467	1.000
CAD, n (%)	31(9.34%)	19(8.84%)	2(4.65%)	0.834	0.359
Prior stroke, n (%)	47(14.20%)	31(14.42%)	3(6.98%)	0.943	0.188
Hcy, μmol/L	14.83 ± 5.44	16.80 ± 7.73	13.71 ± 4.42	**0.001**	**0.011**
Folate level, μg/L	12.17 ± 4.57	10.06 ± 4.98	11.51 ± 3.65	**<0.001**	**0.019**
Vitamin B12 level, ng/L	477.59 ± 186.06	452.85 ± 226.07	456.66 ± 126.83	**0.010**	0.221
Location of aneurysms, n (%)			–	**0.012**	
Anterior circulation artery	309(93.35%)	187(86.98%)			
Posterior circulation artery	22(6.65%)	28(13.02%)			
Multiple aneurysms, n (%)	81(24.47%)	48(22.33%)	–	0.564	
Irregular shape, n (%)	63(19.03%)	142(66.05%)	–	**<0.001**	
Aneurysm diameter, mm	6.19 ± 4.16	5.94 ± 3.04	–	0.473	
eGFR, ml/min/1.73 m^2^	99.88 ± 21.21	113.72 ± 24.55	118.39 ± 26.38	**<0.001**	0.402

*p1: p value between patients with UIAs and those with RIAs. p2: p value between patients with RIAs and those with Na-SAH. UIAs, unruptured intracranial aneurysms; RIAs, ruptured intracranial aneurysms; Na-SAH, non-aneurysmal subarachnoid hemorrhage; CAD, Coronary artery disease; eGFR, estimated glomerular filtration rate; Hcy, homocysteine. The bolded values represent statistical significance, which is defined as data with P-value less than 0.05.*

There were more patients with diabetes mellitus in the UIA group than in the RIA group (16.01 vs. 4.65%, *p* < 0.001). In the UIA group, 309 (93.35%) patients had aneurysms located at the anterior circulation artery, and 22 (6.65%) at the posterior circulation artery. Additionally, 187 (86.98%) aneurysms were located in the anterior circulation artery and 28 (13.02%) at the posterior circulation were ruptured. There was a significant difference in the location of aneurysms (*p* = 0.012). Moreover, aneurysms with irregular shape were more likely to rupture (66.05 vs.19.03%, *p* < 0.001). The result of multivariate logistic regression analysis indicated that after adjusting for confounders, diabetes mellitus (OR 0.403; 95% CI 0.179-0.909, *p* = 0.028), aneurysm location (anterior circulation artery) (OR 0.371; 95% CI 0.175-0.789, *p* = 0.010) and irregular shape (OR 7.339; 95% CI 4.660-11.560, *p* < 0.001) were still independently associated with RIA. There was no significant difference in age, drinking, hypertension, dyslipidemia, CAD, history of stroke, and diameter or number of aneurysms between the UIA and RIA groups.

The proportion of patients with hypertension in RIA group was higher than in Na-SAH group (56.28 vs. 30.23%, *p* = 0.002). After adjusting for confounders, hypertension was still independently associated with RIA (OR 3.490; 95% CI 1.601-7.606, *p* = 0.002). There were no significant differences in demographic characteristics or other complications between the two groups in (all *p* > 0.05).

### Homocysteine Levels of Patients With Unruptured and Ruptured Intracranial Aneurysms

**FIGURE 1:**
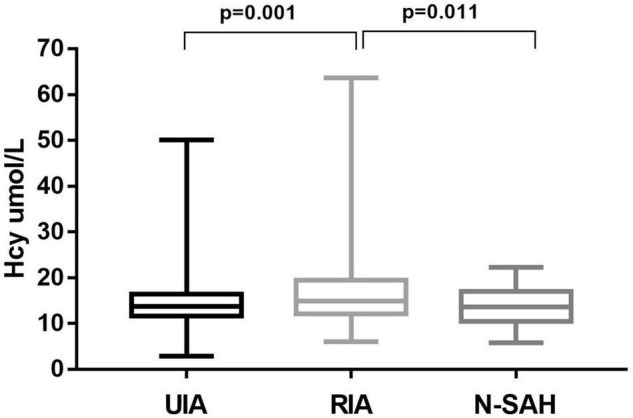
Homocysteine levels of patients with unruptured and ruptured intracranial aneurysms. Hcy levels was higher in patients with RIA than in those with UIA; and in RIA was also higher than that in Na-SAH, the difference was statistically significant (*p* = 0.001 and *p* = 0.011, respectively).

In the univariate analysis (Table 1), Hcy levels were obviously higher in patients with RIA than in those with UIA (16.80 ± 7.73 μmol/L vs.14.83 ± 5.44 μmol/L, *p* = 0.001) (Figure 1). Folate and vitamin B12 levels were lower among those with RIA compared to those with UIA (10.06 ± 4.98 vs. 12.17 ± 4.57 ug/L, *p*<0.001; 452.85 ± 226.07 ng/L vs. 477.59 ± 186.06 ng/L, *p* = 0.010, respectively). In the multivariate logistic regression analysis, the Hcy level was independently associated with the RIA (OR 1.069; 95% CI 1.025–1.114, *p* = 0.002) after adjusting for confounders (Table 2).

**TABLE 2:** Multivariate logistic regression analysis between patients with UIAs and those with RIAs.

Index	OR	95% CI	*P*-value
Sex(female), n (%)	0.902	0.483-1.685	0.747
Smoking, n (%)	0.705	0.327-1.522	0.374
Diabetes mellitus, n (%)	0.403	0.179-0.909	**0.028**
Hcy, μmol/L	1.069	1.025-1.114	**0.002**
Folate level, μg/L	0.945	0.897-0.995	**0.031**
Vitamin B12 level, ng/L	1	1.000-0.999	0.664
eGFR, ml/min/1.73 m^2^	1.029	1.019-1.040	**<0.001**
Location of aneurysms(Anterior circulation artery), n (%)	0.371	0.175-0.789	**0.010**
Irregular shape, n (%)	7.339	4.660-11.560	**<0.001**

*UIA, unruptured intracranial aneurysm; RIA, ruptured intracranial aneurysm; FPG, Fasting plasma glucose; eGFR, estimated glomerular filtration rate; LDL-C, low density lipoprotein cholesterol; Hcy, homocysteine. The bolded values represent statistical significance, which is defined as data with P-value less than 0.05.*

### Homocysteine Levels of Patients With Aneurysmal or Non-Aneurysmal Subarachnoid Hemorrhage

In univariate analysis (Table 1), the Hcy level was significantly higher in patients with RIA (a-SAH) than in those Na-SAH (16.80 ± 7.73 μmol/L vs. 13.71 ± 4.42 μmol/L, *p* = 0.011) (Figure 1). Folate level was lower in RIA (10.06 ± 4.98 μg/L vs. 11.51 ± 3.65 μg/L, *p* = 0.019). There was no significant difference in vitamin B12 levels between the two groups. In the multivariate logistic regression analysis, the Hcy level was independently associated with RIA (OR 1.083; 95% CI 1.002–1.170, *p* = 0.046) after adjusting for confounders (Table 3).

**TABLE 3:** Multivariate logistic regression analysis between patients with RIA and those with Na-SAH.

Index	OR	95% CI	*P*-value
Hcy, μmol/L	1.083	1.002-1.170	**0.046**
Hypertension, n (%)	3.490	1.601-7.606	**0.002**
Folate level, μg/L	0.970	0.897-1.048	0.437

*RIA, ruptured intracranial aneurysm; N-SAH, non-aneurysmal subarachnoid hemorrhage; Hcy, homocysteine. The bolded values represent statistical significance, which is defined as data with P-value less than 0.05.*

## Discussion

This study demonstrated that Hcy levels were higher in patients with RIA than those with UIA. In all patients with SAH, Hcy levels were also higher in patients with aneurysmal SAH than patients with non-aneurysmal SAH. These results suggest that Hcy levels may be related to the rupture of IA.

Many risk factors have been reported to be associated with the rupture of IA, such as female sex, smoking, aneurysm location, aneurysm size, number of aneurysms, and a family history of SAH (Wiebers et al., 2003; Greving et al., 2014; Fuentes et al., 2022; Wang et al., 2022). However, these factors can only explain the risk of rupture of aneurysms to some extent (Kleinloog et al., 2018). The occurrence and development of IAs is a complex process, which may be involved in inflammatory reactions (Andreasen et al., 2013). Considering that Hcy is also a biomarker of inflammatory response, it may have an effect on the aneurysms. Animal experiments have proven that HHcy accelerates cerebral aneurysm formation in a rat model (Xu et al., 2011). Several recent observational studies have found that Hcy levels in patients with IAs were significantly higher than those in non-aneurysmal patients (Ren et al., 2017; Rosi et al., 2018; Wang et al., 2020). This is consistent with our findings. However, whether Hcy levels are associated with aneurysm rupture has not received enough attention.

Our study found that Hcy levels in patients with RIA were significantly higher than those in patients with UIA, suggesting that Hcy may be one of the risk factors for IA rupture. Concurrently, to rule out the interference of intracranial hemorrhage on Hcy, we further compared a-SAH and Na-SAH patients to SAH. The results confirm our supposition that Hcy in the a-SAH group was both significantly higher and independently correlated. To our knowledge, this is the first clinical study to investigate the relationship between Hcy levels and IA rupture in the population. Previous animal experiments suggested that Hcy could be related to the rupture of IAs (Xu et al., 2011; Korai et al., 2016). In detail, Hcy can activate a series of complex processes, including decreasing arterial reactivity to vasomotor stimuli, increasing the production of free oxygen radicals, and stimulating the proliferation of smooth muscle cells in the arterial wall (Evers et al., 1997; Eikelboom et al., 2000). All these processes lead to wall injury, which plays an important role in the rupture of IAs (Andreasen et al., 2013). This may provide a new intervention factor for the prevention of aneurysm rupture.

In addition, we found that the location of aneurysms and irregular shape of IA were also associated with RIA, which was consistent with previous studies (Greving et al., 2014; Fuentes et al., 2022; Wang et al., 2022). Future studies with larger sample sizes are needed to assess other associated factors.

Our study has several strengths. First, we compared Hcy in UIA and RIA groups, and a-SAH and Na-SAH groups, respectively, which has rarely been assessed in previous studies. Therefore, the conclusion in our study provides strong evidence of the role of Hcy. Second, the large sample size provided considerable statistical power, which likely contributed to detecting real differences between UIA and RIA. Third, all patients underwent a DSA, which is superior due to its high resolution and better detection of IAs (Kwak et al., 2020). The comprehensive information of each patient in the study ensured clear risk factors and results. Moreover, IAs were repeatedly excluded by CT or DSA in patients with Na-SAH, and our grouping conditions were strict and precise.

However, the present study had some limitations. First, this was a cross-sectional study. Therefore, the causal relationship between Hcy and the rupture of IA needs confirmation from a longitudinal study. Second, the study was a retrospective study conducted in a single center, which inevitably resulted in selection bias. Future multicenter prospective cohort studies are needed to confirm this conclusion. Third, although many factors that may affect Hcy levels were included in this study, some were not included in the analysis, such as MTHFR gene mutations, which have been found to cause high Hcy levels (Feng et al., 2021).

## Conclusion

In conclusion, homocysteine levels are associated with the rupture of IA. These findings provide novel insights into IA rupture. Future studies are needed to confirm this relationship and determine whether controlling HHcy with dietary changes, or folate and Vit B12 supplementation may prevent IA rupture.

## Data Availability Statement

The raw data supporting the conclusions of this article will be made available by the authors, without undue reservation.

## Ethics Statement

The studies involving human participants were reviewed and approved by Ethics committee of the First Affiliated Hospital of Zhengzhou University. The patients/participants provided their written informed consent to participate in this study.

## Author Contributions

SW designed the whole study, collected and analyzed the data. XY drafted the manuscript. DL and XG contributed to the discussion and analyzed the data. SG and YX critically reviewed manuscript for important intellectual content. All authors contributed to the article and approved the submitted version.

## Conflict of Interest

The authors declare that the research was conducted in the absence of any commercial or financial relationships that could be construed as a potential conflict of interest.

## Publisher’s Note

All claims expressed in this article are solely those of the authors and do not necessarily represent those of their affiliated organizations, or those of the publisher, the editors and the reviewers. Any product that may be evaluated in this article, or claim that may be made by its manufacturer, is not guaranteed or endorsed by the publisher.

## References

[B1] Andreasen T. H. Bartek J. Jr. Andresen M. Springborg J. B. Romner B. (2013). Modifiable risk factors for aneurysmal subarachnoid hemorrhage. *Stroke* 44 3607–3612. 10.1161/STROKEAHA.113.001575 24193807

[B2] Eikelboom J. W. Hankey G. J. Anand S. S. Lofthouse E. Staples N. Baker R. I. (2000). Association between high homocyst(e)ine and ischemic stroke due to large- and small-artery disease but not other etiologic subtypes of ischemic stroke. *Stroke* 31 1069–1075. 10.1161/01.str.31.5.1069 10797167

[B3] Evers S. Koch H. G. Grotemeyer K. H. Lange B. Deufel T. Ringelstein E. B. (1997). Features, symptoms, and neurophysiological findings in stroke associated with hyperhomocysteinemia. *Arch. Neurol.* 54 1276–1282. 10.1001/archneur.1997.00550220074017 9341574

[B4] Feng W. Zhang Y. Pan Y. Zhang Y. Liu M. Huang Y. (2021). Association of three missense mutations in the homocysteine-related MTHFR and MTRR gene with risk of polycystic ovary syndrome in Southern Chinese women. *Reprod. Biol. Endocrinol.* 19:5. 10.1186/s12958-020-00688-8 33407572 PMC7789417

[B5] Fuentes A. M. Stone McGuire L. Amin-Hanjani S. (2022). Sex differences in cerebral aneurysms and subarachnoid hemorrhage. *Stroke* 53 624–633. 10.1161/STROKEAHA.121.037147 34983239

[B6] Greving J. P. Wermer M. J. Brown R. D. Jr. Morita A. Juvela S. Yonekura M. (2014). Development of the PHASES score for prediction of risk of rupture of intracranial aneurysms: a pooled analysis of six prospective cohort studies. *Lancet Neurol.* 13 59–66. 10.1016/S1474-4422(13)70263-1 24290159

[B7] Jacques P. F. Bostom A. G. Wilson P. W. Rich S. Rosenberg I. H. Selhub J. (2001). Determinants of plasma total homocysteine concentration in the Framingham Offspring cohort. *Am. J. Clin. Nutr.* 73 613–621. 10.1093/ajcn/73.3.613 11237940

[B8] Kleinloog R. de Mul N. Verweij B. H. Post J. A. Rinkel G. J. E. Ruigrok Y. M. (2018). Risk factors for intracranial aneurysm rupture: a systematic review. *Neurosurgery* 82 431–440. 10.1093/neuros/nyx238 28498930

[B9] Korai M. Kitazato K. T. Tada Y. Miyamoto T. Shimada K. Matsushita N. (2016). Hyperhomocysteinemia induced by excessive methionine intake promotes rupture of cerebral aneurysms in ovariectomized rats. *J. Neuroinflamm.* 13:165. 10.1186/s12974-016-0634-3 27349749 PMC4924228

[B10] Kwak Y. Son W. Kim Y. S. Park J. Kang D. H. (2020). Discrepancy between MRA and DSA in identifying the shape of small intracranial aneurysms. *J. Neurosurg.* 134 1887–1893. 10.3171/2020.4.JNS20128 32707543

[B11] Levey A. S. Bosch J. P. Lewis J. B. Greene T. Rogers N. Roth D. (1999). A more accurate method to estimate glomerular filtration rate from serum creatinine: a new prediction equation. Modification of diet in renal disease study group. *Ann. Intern. Med.* 130 461–470. 10.7326/0003-4819-130-6-199903160-00002 10075613

[B12] Li J. G. Chu J. Barrero C. Merali S. Praticò D. (2014). Homocysteine exacerbates β-amyloid pathology, tau pathology, and cognitive deficit in a mouse model of Alzheimer disease with plaques and tangles. *Ann. Neurol.* 75 851–863. 10.1002/ana.24145 24644038 PMC4362695

[B13] Li T. Yu B. Liu Z. Li J. Ma M. Wang Y. (2018). Homocysteine directly interacts and activates the angiotensin II type I receptor to aggravate vascular injury. *Nat. Commun.* 9:11. 10.1038/s41467-017-02401-7 29296021 PMC5750214

[B14] Miao Y. Zhao Y. Han L. Ma X. Deng J. Yang J. (2021). NSun2 regulates aneurysm formation by promoting autotaxin expression and T cell recruitment. *Cell Mol. Life Sci.* 78 1709–1727. 10.1007/s00018-020-03607-7 32734582 PMC11073013

[B15] Pařízková M. Andel R. Lerch O. Marková H. Gažová I. Vyhnálek M. (2017). Homocysteine and real-space navigation performance among non-demented older adults. *J. Alzheimers Dis.* 55 951–964. 10.3233/JAD-160667 27802238

[B16] Ren J. R. Ren S. H. Ning B. Wu J. Cao Y. Ding X. M. (2017). Hyperhomocysteinemia as a risk factor for saccular intracranial aneurysm: a cohort study in a chinese han population. *J. Stroke Cerebrovasc. Dis.* 26 2720–2726. 10.1016/j.jstrokecerebrovasdis.2017.01.001 28943219

[B17] Rosi J. Morais B. A. Pecorino L. S. Oliveira A. R. Solla D. J. F. Teixeira M. J. (2018). Hyperhomocysteinemia as a risk factor for intracranial aneurysms: a case-control study. *World Neurosurg.* 119 e272–e275. 10.1016/j.wneu.2018.07.132 30053565

[B18] Selhub J. Jacques P. F. Wilson P. W. Rush D. Rosenberg I. H. (1993). Vitamin status and intake as primary determinants of homocysteinemia in an elderly population. *JAMA* 270 2693–2698. 10.1001/jama.1993.03510220049033 8133587

[B19] Shi Z. Guan Y. Huo Y. R. Liu S. Zhang M. Lu H. (2015). Elevated total homocysteine levels in acute ischemic stroke are associated with long-term mortality. *Stroke* 46 2419–2425. 10.1161/STROKEAHA.115.009136 26199315 PMC4542568

[B20] Shoamanesh A. Preis S. R. Beiser A. S. Kase C. S. Wolf P. A. Vasan R. S. (2016). Circulating biomarkers and incident ischemic stroke in the Framingham Offspring Study. *Neurology* 87 1206–1211. 10.1212/WNL.0000000000003115 27558379 PMC5035987

[B21] Tawk R. G. Hasan T. F. D’Souza C. E. Peel J. B. Freeman W. D. (2021). Diagnosis and treatment of unruptured intracranial aneurysms and aneurysmal subarachnoid hemorrhage. *Mayo Clin. Proc.* 96 1970–2000. 10.1016/j.mayocp.2021.01.005 33992453

[B22] Vergouwen M. D. Jong-Tjien-Fa A. V. Algra A. Rinkel G. J. (2016). Time trends in causes of death after aneurysmal subarachnoid hemorrhage: a hospital-based study. *Neurology* 86 59–63. 10.1212/WNL.0000000000002239 26590269

[B23] Vlak M. H. Algra A. Brandenburg R. Rinkel G. J. (2011). Prevalence of unruptured intracranial aneurysms, with emphasis on sex, age, comorbidity, country, and time period: a systematic review and meta-analysis. *Lancet Neurol.* 10 626–636. 10.1016/S1474-4422(11)70109-0 21641282

[B24] Wang J. Weng J. Li H. Jiao Y. Fu W. Huo R. (2021). Atorvastatin and growth, rupture of small unruptured intracranial aneurysm: results of a prospective cohort study. *Ther. Adv. Neurol. Disord.* 9:1756286420987939. 10.1177/1756286420987939 33953800 PMC8042545

[B25] Wang Q. Zhang J. Zhao K. Xu B. (2020). Hyperhomocysteinemia is an independent risk factor for intracranial aneurysms: a case-control study in a Chinese Han population. *Neurosurg. Rev.* 43 1127–1134. 10.1007/s10143-019-01138-9 31256274

[B26] Wang Y. Cheng M. Liu S. Xie G. Liu L. Wu X. (2022). Shape related features of intracranial aneurysm are associated with rupture status in a large Chinese cohort. *J. Neurointerv. Surg.* 14 252–256. 10.1136/neurintsurg-2021-017452 33883209

[B27] Wiebers D. O. Whisnant J. P. Huston J. III Meissner I. Brown R. D. Jr. Piepgras D. G. (2003). Unruptured intracranial aneurysms: natural history, clinical outcome, and risks of surgical and endovascular treatment. *Lancet* 362 103–110. 10.1016/s0140-6736(03)13860-3 12867109

[B28] Xu Y. Tian Y. Wei H. J. Dong J. F. Zhang J. N. (2011). Methionine diet-induced hyperhomocysteinemia accelerates cerebral aneurysm formation in rats. *Neurosci. Lett.* 494 139–144. 10.1016/j.neulet.2011.02.076 21382440

